# Inequalities in Children's Experiences of Home Learning during the COVID‐19 Lockdown in England*

**DOI:** 10.1111/1475-5890.12240

**Published:** 2020-11-30

**Authors:** Alison Andrew, Sarah Cattan, Monica Costa Dias, Christine Farquharson, Lucy Kraftman, Sonya Krutikova, Angus Phimister, Almudena Sevilla

**Affiliations:** ^1^ Institute for Fiscal Studies; University College London; ^2^ Institute for Fiscal Studies; ^3^ University of Bristol; Institute for Fiscal Studies; University of Porto; ^4^ UCL Institute of Education

**Keywords:** COVID‐19, education, home learning, inequality

## Abstract

This paper combines novel data on the time use, home‐learning practices and economic circumstances of families with children during the COVID‐19 lockdown with pre‐lockdown data from the UK Time Use Survey to characterise the time use of children and how it changed during lockdown, and to gauge the extent to which changes in time use and learning practices during this period are likely to reinforce the already large gaps in educational attainment between children from poorer and better‐off families. We find considerable heterogeneity in children's learning experiences – amount of time spent learning, activities undertaken during this time and availability of resources to support learning. Concerningly, but perhaps unsurprisingly, this heterogeneity is strongly associated with family income and in some instances more so than before lockdown. Furthermore, our analysis suggests that any impacts of inequalities in time spent learning between poorer and richer children are likely to be compounded by inequalities not only in learning resources available at home, but also in those provided by schools.

## I. Introduction

The school closures that have been imposed around the world to reduce the spread of the coronavirus are one of the most defining features of the COVID‐19 crisis. On 20 March 2020, UK schools closed their gates to all but the children of essential workers and those deemed most vulnerable. The majority of children then spent more than a full term out of school.

Months out of school risk setting back children's learning and development.^1^ This is particularly concerning for children from disadvantaged backgrounds, who already achieve less well on average than their better‐off classmates.^2^ By bringing home all education investments, the pandemic is reducing the equalising role that the time children normally spend in school may have in their learning. Combined with the disproportionate effect of the crisis on the finances and employment of poorer households, the COVID‐19 crisis could have disastrous consequences for inequalities in children's educational attainment.^3^

In this paper, we use newly collected data on the time use, home‐learning practices and financial circumstances of families to study the learning experiences of children during lockdown. Specifically, we focus on education investments that have been shown to matter in the long term for educational attainment, such as the time that children spend in classes (which happened online during this period), doing school work or studying with a tutor, as well as the home‐learning resources they have available to support their studying time, including those made available by schools and families. We combine these data with pre‐lockdown information from the 2014–15 UK Time Use Survey to examine how the lockdown affected education investments and inequalities therein.

Our unique real‐time data provide an opportunity to examine how children were spending their time under lockdown in more depth than would be possible using data collected retrospectively. Characterising these experiences of home learning and how they differ from regular school‐based learning is crucial to understand how children have lived through this period and to anticipate the long‐lasting consequences this disruption may have for their educational attainment. Our aim is to characterise inequalities in home learning and how they relate to pre‐existing inequalities in ways that may reinforce or attenuate differences in the educational attainment of children. Understanding the formation of learning inequalities during lockdown is crucial to inform policy aimed at supporting families with children and schools during and after this period, and this paper provides a first step in that direction.

Existing evidence offers some important insights into the mechanisms through which lockdown may have affected children's learning. First, and perhaps most obviously, school closures have removed most children from their physical school environment for over a term – around 40 per cent of a regular school year. Several studies suggest that the loss of instructional time – delivered by teachers working to the national curriculum on which children will eventually be examined – is likely to create substantial learning losses. For example, Lavy (2015) looks across 50 countries and finds that an extra hour of instructional time each week in the main subjects increases test scores by around 6 per cent of a standard deviation. Other studies corroborate these findings by documenting the negative effects of reductions in instructional time due to a policy reform^4^ and of time away from school during summer holidays^5^ or due to inclement weather^6^ on education outcomes.

The extent to which the loss of school‐based instructional time and other school‐based programmes will harm students' outcomes and inequalities between them depends on how home learning is implemented in each school and in each family. Existing studies suggest that additional time spent with parents can have positive effects on child development, especially among younger children.^7^ Evidence across several contexts including the UK, the US and Australia shows that this effect is driven by time spent on educationally oriented and structured activities rather than unstructured and passive time with parents.^8^ What parents do with the large amount of extra time with their children during this crisis is therefore likely to have significant effects on the children's development.

Even if more time with parents will counterbalance some of the adverse effects of loss of instructional time, it is unlikely to do so equally for all children. There is some evidence that the positive effects of time spent with parents are stronger for children of more educated parents.^9^ Consistent with these results, Slates et al. (2012) and Alexander, Pitcock and Boulay (2016) show that the accumulated disadvantage from the summer holiday period may account for two‐thirds of the attainment gap between the richest and poorest children. School closure during the summer can increase financial pressure and food insecurity in households and reduce access to stimulating activities, which may disproportionately affect disadvantaged children.^10^

The developmental benefits of parental time might also have changed during the lockdown, potentially differentially between families. There is a large literature that links parental well‐being and stress to children's outcomes. These impacts can be felt from mothers who are stressed while pregnant,^11^ in childhood^12^ and in adolescence.^13^ This is sobering evidence in light of the current situation; families, particularly low‐income families, are facing adverse economic and health shocks. Indeed, several studies have found that the lockdown has had a particularly negative impact on the mental health and well‐being of women of childbearing age.^14^ At the same time, children are spending so much more time with their parents and have little access to their wider social and support networks, which in ‘normal’ times might play a protective role.

At this early stage, we are not able to study causal impacts of lockdown on children's learning or test any of these possible mechanisms. However, the evidence we present in this paper suggests that children are at considerable risk of suffering from significant long‐term adverse consequences of lockdown, especially those from low‐income families. In the absence of significant policy intervention in the short term, these risks are likely to become a reality. Our results show, predictably, the shift of learning time from school to home, with considerable heterogeneity in the amount of time children spent learning, what activities they did during this time and what resources they had to support their learning. We find that this heterogeneity is strongly associated with family income; in some instances, these socio‐economic gradients have increased during the crisis. Furthermore, our analysis suggests that the adverse effects of inequalities in time spent learning between poorer and richer children are likely to be compounded by inequalities not only in resources available at home that children need to make the most of their learning time, but also in those provided by schools.

Section II describes the data that we collected and how our study sample compares with a nationally representative sample. Section III presents a snapshot of children's time use before and during lockdown on the weekday preceding the day in which the survey was completed, as well as a breakdown of time spent learning into different types of learning activities. We then focus on differences in learning time across children from lower‐ and higher‐income families in Section IV. Section V extends this analysis to also look at differences in the home‐learning resources available to children by family income and the degree to which these mediate any income gradients in learning time during lockdown. Section VI concludes.

## II. Data

To analyse how the lockdown is affecting the time use of children and their learning activities, we use two main sources of data. The first is unique real‐time data that we collected through an online survey of families of school‐aged children living in England in the first two months of the lockdown. The second source is the 2014–15 UK Time Use Survey.^15^

### 1. Real‐time survey of time use during the lockdown

We surveyed 5,582 parents living in England with at least one child aged 4–15 and in year group Reception, 1, 4, 5, 8, 9 or 10.^16^ These are year groups that will, in the next year or two, take one of the standardised national assessments. Our sample was constructed to allow us to link in information about those assessments from administrative data sources in the future, once it becomes available, to study the longer‐term impact of this crisis on children's learning. However, the sample also provides important insights into how lockdown and school closures affected what children of different ages and from different families were doing during the 2020 summer term, which is the focus of this paper.

Data collection ran over the period 29 April to 20 June 2020.^17^ The survey gathered detailed information on how children spent their time on a term‐time weekday. One parent per family was asked to fill in an online time diary for one of their (randomly selected) children aged 4–15, telling us what activities they did during each hour of the previous day and who they were with. Interviews were conducted on Tuesdays to Saturdays (excluding the days after public holidays) to ensure that the information refers to ‘school’ days. We also collected rich information about the types of home‐learning activities children were doing and the resources they had available for supporting their learning, including those provided by the school and the facilities at home. These data are complemented with detailed demographic and socio‐economic information about the family, including on the working status and income of the parents before and during the crisis.

To ensure the representativeness of our sample, we imposed sampling quotas based on a number of characteristics, including the gender, education and pre‐lockdown employment status of the responding parent and the region of residence of the family. We worked with a reputable online survey company to stratify the sample and ensure it represents diversity in the population along these dimensions.

Predictably, this procedure did not produce a balanced sample along additional important characteristics relative to the population of families with children in England. The first and third columns of Table A1 in the online appendix show how the distributions of some socio‐economic characteristics in our sample compare with those found in the Labour Force Survey (LFS) for 2019, implemented on a representative large sample of the population. The comparison reveals that our sample tends to be better educated and from higher economic strata than the population, which is likely to reflect the fact that the survey was voluntary and conducted online.

To correct for sampling bias, we constructed a subsample from the LFS on criteria similar to our selection rules for surveying families. We used that sample to construct balancing weights for our sample on many characteristics, including parental education, their pre‐lockdown working status and income, and the types of industry and occupations they worked in, as well as region of residence. The second column of Table A1 shows the means of these variables in our now weighted survey sample and confirms that our reweighted sample reproduces closely the distribution of these characteristics in the LFS for 2019.

### 2. UK Time Use Survey

In this paper, we use the most recent (2014–15) round of the UK Time Use Survey (UKTUS) as a second source of data in order to compare time use during and before lockdown. The UKTUS is a diary‐based time use survey of a representative sample of 4,238 households across all four nations of the UK. The survey captures diary information for two randomly selected days of the week, one a weekday and one on a weekend, for all household members aged 8 and above. Each respondent recorded what they were doing in each 10‐minute slot of the day, as well as where they were and with whom. This is the gold standard in time‐use diary data collection and processing and delivers a very detailed description of use of time. However, this method is very burdensome on respondents and interviewers, as how to fill out the diary needs to be explained. Similarly, coders need to be trained on how to process the diary open‐text answers, and quality checks need to be put in place so that verbatim responses can be coded into activities. As a result, processing the data to make them readable becomes much slower and costlier than with online surveys where activities are pre‐coded.^18^

In this paper, we utilise data from the UKTUS on time use of children aged between 8 and 15 on school days, alongside family composition, number and age of children, and family earnings. We use weights provided by the survey to ensure the representativeness of the data. In order to maximise comparability between the sample in our survey and the UKTUS, we exclude children under the age of 8 from our sample in this paper. We now describe the main measures that we use in the analysis.

### 3. Socio‐economic status

A big focus in this paper is the differences in children's experiences during the lockdown across families in different socio‐economic groups. In our survey, we measure socio‐economic background using the family's pre‐tax annual earnings in 2019. We equivalise this measure to best reflect the amount of resources available to household members, accounting for the fact that bigger families need higher incomes to enjoy the same standard of living and that adults typically require more resources than children.^19^ We construct a comparable measure for the UKTUS sample by equivalising reported household earnings in 2014–15 to account for household size in the same way. In what follows, we use family income and family earnings interchangeably.

### 4. Time use of children

In order to capture time use, we asked respondents to fill in information about what the selected school‐aged child in the household was doing in each one‐hour slot in the previous 24 hours. Respondents could choose from the following activities: sleeping, personal care, learning, at school, reading, playing outdoors, playing indoors, socialising, on a screen, other hobbies and housework. For each, we provided brief explanations and examples of what falls in that group. For adolescents, we also allowed the parent respondent to say they did not know what the child was doing during a particular hour. Since children may spend less than an hour on a given activity, we allowed respondents to report that a child was doing more than one activity within each one‐hour time slot. Our data thus capture *the number of one‐hour slots* during which children were reported doing a particular activity. While our time diaries do not allow us to determine precisely *how long* children spent on a particular activity, and in particular may provide upward‐biased measures of time spent in each activity, they do reveal sensible patterns that are well aligned with those reported by the UKTUS.

We further complemented our time diaries with questions on the number of hours a week that children spent on four specific learning‐related tasks over the course of a typical week during the lockdown. These tasks included online classes provided by the school, any other work set by the school (for example, home‐learning packs), being with a paid private tutor, and other educational activities. These data allow for more in‐depth analysis of the amount of time children spent on key home‐learning activities.

In the UKTUS, we aggregate time use categories to construct measures that align with those in our survey. When comparing our time use data with those from 2014–15, we use the 10‐minute slots in the UKTUS to construct indicators for whether the child spent any time doing the activity in a particular hour and add up these indicators to measure the number of hourly time slots in which a given activity was recorded. We only include information from surveys conducted on school or college days. When comparing our data on learning time (based on parental report of total hours spent on specific learning activities over a typical week) with UKTUS data, we aggregate the information from the 10‐minute slots in the UKTUS to measure the number of minutes spent on that learning activity per day.^20^

## III. Children's time use before and during lockdown

**FIGURE 1:**
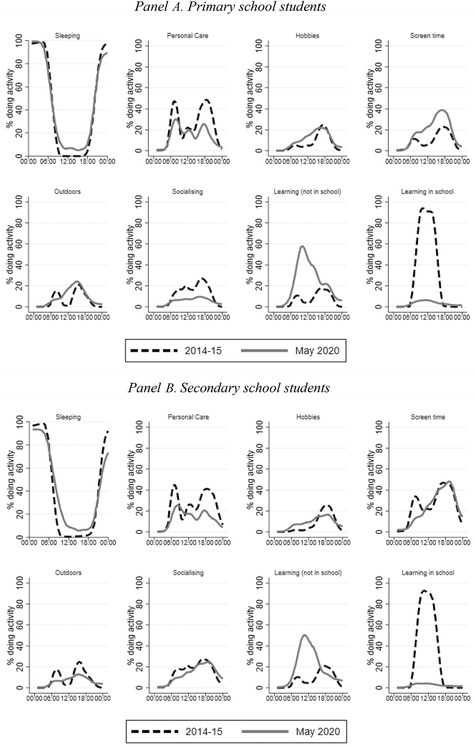
Children's and adolescents' activities over the course of a ‘school’ day during lockdown *Note*: Lines show the percentage of individuals reporting doing each activity in each hour for the UKTUS (2014–15) and the COVID survey (May 2020). All lines are smoothed using Epanechnikov kernels. In the UKTUS, an activity is coded as having taken place if any time is spent on that activity in that hour.

We start by providing an overview of how children were spending their time in lockdown and compare these patterns with those observed a few years before the lockdown, in 2014–15. Figure 1 presents sequences of activities in which children were engaged in each hour over the course of the day, with Panels A and B showing results for primary and secondary school children respectively. For comparability, we use information aggregated in hourly time slots for both surveys. Figures based on the more detailed 10‐minute slots available for the UKTUS are shown in Figure A1 in the online appendix. They show patterns that are very similar to (although predictably more granular than) those plotted in Figure 1, suggesting that any bias resulting from the hour slot aggregation is likely to be small.

Figure 1 shows some predictable patterns. For instance, sleeping time was broadly unaffected by lockdown: school‐aged children sleep throughout the night, with secondary school children going to sleep later (especially during the lockdown) and waking up later than primary school children. Before and during lockdown, other personal care is most likely to take place after waking up and before going to bed. These patterns are sensible, suggesting that our data are capturing daily activities in ways that are consistent with those in state‐of‐the‐art time use surveys such as the UKTUS.

The rest of the children's day is filled with learning and leisure activities. While before lockdown primary and secondary school children spent roughly the same amount of time socialising, there was a big drop in socialising for primary school children in May and June 2020. This is likely a direct result of the social distancing measures, which closed down playgrounds and ruled out play dates. Interestingly, older children are socialising just as much as they used to, most likely reflecting the importance of virtual social connections for this age group. In contrast, there was a noticeable decline in the proportion of secondary school children spending time outside throughout the day which is not there for primary school children. Screen time increased for primary school children at all points in the day, less so for secondary school children, who were already spending more time on screens than primary school children before lockdown.

Figure 1 shows that education time shifted from school to being fully concentrated at home for children of all ages. It also shows a decline in the proportion of children engaged in learning‐related activities at the times of the day when they used to be most prevalent pre‐lockdown. A maximum of 60 per cent of children were engaging in learning activities in a given hour during lockdown; before the lockdown, the maximum was over 90 per cent. Together this amounts to children spending less time overall in learning activities during lockdown than they used to.

This is also what we find when we look at data on mean hours spent on learning activities during and before lockdown. Here we move away from learning time reported in time‐use diaries and instead use an alternative measure, based on parental reports of hours spent in a typical week on different home‐learning activities, which is more comparable to pre‐lockdown measures in the UKTUS.^21^ Table 1 shows that, in line with Figure 1, average total time in which some learning took place (at school and outside school) decreased from 6.3 hours before lockdown to 4.1 hours during lockdown. The drop was even bigger for secondary school pupils, from 6.59 to 4.15 hours.

**TABLE 1:** Average total learning time on ‘school’ days before and during lockdown

	*Primary*	*Secondary*	*Total*
During lockdown: recall questions	4.06	4.15	4.1
Before lockdown	5.99	6.59	6.3

*Note*: Recall question measure of learning time during lockdown is calculated by dividing by 5 total time reported in recall questions about time spent on educational activities in a typical lockdown week. We consider observations where more than 12 hours of learning per day were reported as outliers and exclude them from the analysis. This was the case for 6.9 per cent of the observations. The ‘before lockdown’ measure of learning time is calculated by summing 10‐minute slots in which children report learning in the UKTUS 2014–15 data. For comparability with recall lockdown measures, we only include children in the UKTUS sample who report attending school for at least some of the time.

While the evidence we have shown so far clearly points to the fact that children were, on average, spending less time engaging in learning activities during lockdown, it does not tell us about differences in the experiences of learning during lockdown. Before lockdown, the vast majority (90 per cent) of all learning activities were carried out in school, in close contact with teachers. During lockdown, however, the time children dedicate to different learning activities is likely to be much more varied and dependent on the resources available to support their learning.

**FIGURE 2:**
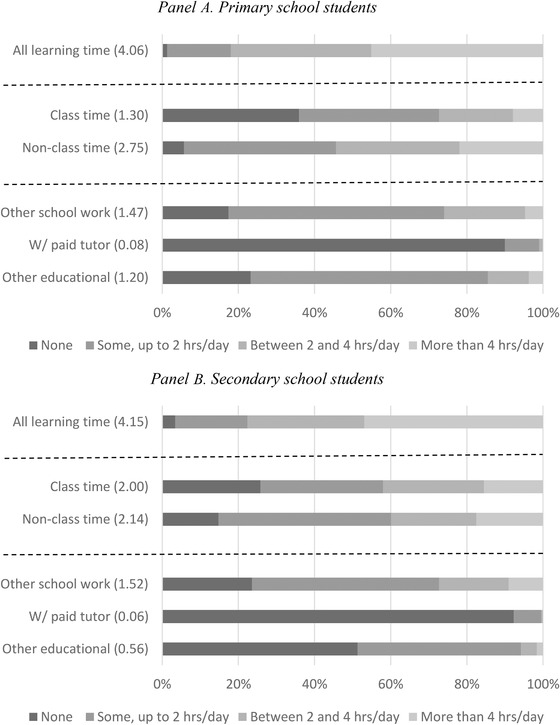
Distribution of time spent on educational activities on a ‘school’ day (from recall questions) *Note*: These statistics are computed for a sample of children including those who did not attend school the day before their parents answered the survey, who have valid time‐diary responses and non‐missing answers to the recall questions, and whose total learning time was less than or equal to 12 hours. ‘All learning time’ is the sum of class and non‐class time. ‘Non‐class time’ is the sum of ‘other school work’, ‘w/ paid tutor’ and ‘other educational’. Numbers in parentheses are average hours spent.

To investigate this, we turn to examining what children were doing during *learning* time in lockdown, using parental reports on time spent on online classes, on other school work, with a paid tutor or doing other educational activities, during a typical lockdown week, and how much variation there was in this across children. First, Figure 2 shows that there were substantial differences in how much time children were spending on any type of learning during lockdown: around a third of children of all ages spent between 2 and 4 hours per day on learning activities and nearly a half spent more than 4 hours. There is, however, a non‐negligible minority of around 20 per cent of secondary school children and slightly fewer primary school children who did less than 2 hours per day.

Beyond total learning time, the type of activities conducted is important – some activities are more conducive to learning than others.^22^ Online classes are likely the closest substitute to a regular class structure that children would have experienced pre‐lockdown. On average, online classes account for 1.3 and 2 hours of the school days of children in primary and secondary schools respectively, which is a much shorter time than the time taken by regular classes in a normal school day. However, these averages mask large variation; while 36 per cent and 26 per cent of primary and secondary school students were reported to spend 0 hours doing online classes respectively, over a quarter of primary school children and over 40 per cent of secondary school children do more than 2 hours.

Outside of online classes, primary and secondary school children spend over 2 hours a day on learning (‘non‐class time’). Once again, there is large variation in this dimension of time use, but around 40 per cent of primary and 60 per cent of secondary school children do 2 or fewer hours. During this time, less than 10 per cent of children were spending time with a paid tutor during the week; among those who did, the average time spent with a tutor was under 1 hour a week. There is also a large majority of primary school children spending some time on other educational activities outside those for school and tutoring; in contrast, around half of secondary school children do not report any time on this category. While it is difficult to interpret these patterns without further information on what the activities include, these patterns could potentially reflect the fact that the work set by primary schools takes less time to do than the work set by secondary schools, leading the parents of primary school children to feel the need to keep their children busy with additional learning activities.

## IV. Socio‐economic differences in home learning

Having provided a broad overview of children's time use during lockdown, we now consider what these patterns may mean for evolution of future educational inequalities between children from higher‐ and lower‐income families. There is mounting evidence that large socio‐economic gaps in education investments are key drivers of the also large socio‐economic gaps in education attainment.^23^ There is a real risk that this crisis will widen gaps in attainment further by reducing the equalising role that the time children spend learning at school rather than at home is likely to play. To investigate this, we start by looking at whether there was a change in the socio‐economic gradient in time spent learning as a result of the lockdown. We then study inequalities in the resources that children have available to support their learning at home in Section V.

We quantify the impact of the lockdown on the economic gradient in time spent learning by combining our data with UKTUS data to estimate the following simple two‐period regression model:

(1) $$Y_{i}=\;\alpha +\beta Post_{i}+\gamma Income_{i}+\delta Income_{i}\times Post_{i}+\eta X_{i}+\;\varepsilon_{i},$$

where the subscript i identifies the child. Yis time spent learning, which is computed from the 10‐minute slot records in the UKTUS and compared with the recall measures of our survey. The regression includes the indicator variable Post, which equals 1 if the observation is from our sample of the post‐lockdown period and 0 if it is from the UKTUS sample of 2014–15; the variable Income, which denotes the family's rank in the distribution of equivalised gross parental earnings in the pooled sample; and the interaction Income×Post. The parameter γ measures the relation between family income rank and learning time before the lockdown, and the coefficient δ measures by how much the lockdown changed that relationship. Finally, we also control for a vector of other covariates, $X_{i}$, which include age (in years) dummies, number of siblings, and indicators for whether the child lives in a lone‐parent household and whether he/she is the oldest.^24^ Note that the family's income rank is on a scale of 0 to 1, so coefficients associated with the variable refer to the effect associated with going up from the very bottom to the very top of the income distribution.

For comparability with the UKTUS and expositional clarity, we use the learning time categories presented in Figure 2: ‘class time’, ‘non‐class time’ and ‘total time’. Class time includes time spent in online classes in our survey, and time spent in classes at school/college (including short breaks but not lunch breaks and free periods) in UKTUS.^25^ Non‐class time includes time spent with a paid tutor, on other school work and on other educational activities in our survey, and time spent on homework and other free‐time study (including extracurricular activities such as art and music) in UKTUS. Total time combines class and non‐class time.

Table 2 shows estimates of the income gradient separately for primary and secondary school children for each of these three groups of activities. Columns 1–3 suggest that the learning time of primary school children was not associated with family income prior to lockdown. That holds for all learning time in column 1 and both class and non‐class learning time in columns 2 and 3. This might not be surprising given that most learning activities of young children happen in school, and the length of school days in primary school varies little from school to school. However, the third row of the table shows that family income matters much more during lockdown, with differences in total learning time of nearly 1.5 hours a day between a child at the bottom and a child at the top of the income distribution. For instance, the estimates in column 1 mean that a child in the 10^th^ percentile of the family income distribution does about 35 minutes less of learning time per day than her peer in the median‐income family, and 1 hour 10 minutes less than her peer in the 90^th^ percentile. Moreover, columns 2 and 3 show that family income has a larger impact on time spent in (online) classes than on other ‘non‐class’ learning activities.

**TABLE 2:** Effect of lockdown on inequalities in learning time by income

	*(1)*	*(2)*	*(3)*	*(4)*	*(5)*	*(6)*
	Primary school students			Secondary school students		
	*Total learning time*	*Class time*	*Non‐class time*	*Total learning time*	*Class time*	*Non‐class time*
Lockdown	−2.233***	−4.639***	2.616***	−2.070***	−3.680***	1.785***
	(0.307)	(0.253)	(0.169)	(0.259)	(0.198)	(0.196)
Income rank	0.124	0.135	−0.00963	1.221***	0.852***	0.379***
	(0.145)	(0.121)	(0.0806)	(0.152)	(0.118)	(0.117)
Income rank × Lockdown	1.468***	1.142***	0.500*	0.162	0.0145	0.635*
	(0.507)	(0.416)	(0.280)	(0.436)	(0.333)	(0.330)
Constant	5.733***	5.488***	0.249***	5.727***	5.276***	0.439***
	(0.149)	(0.125)	(0.0828)	(0.147)	(0.114)	(0.112)
Observations	1,256	1,298	1,265	1,794	1,863	1,826
R^2^	0.077	0.389	0.405	0.140	0.358	0.165

*Note*: These coefficients are ordinary least squares (OLS) estimates of equation 1, which also controls for child's age (in years) dummies, number of siblings living in the household, and indicators for lone‐parent household and for whether the child is the oldest. All regressions are weighted using the procedure described in Section II. Standard errors are given in parentheses. ^***^ *p*<0.01, ^**^ *p*<0.05, ^*^ *p*<0.1.

The results for secondary school children show a different pattern. For them, there is a much clearer association between income and time spent on class and non‐class learning activities before lockdown. That association could be partly due to some parents reporting extracurricular activities performed at school after core school hours as ‘classes and lectures’ in the UKTUS (which would mean that these are included in the ‘class time’ category) and better‐off children being more likely to engage in these activities than their less affluent peers.

Lockdown did little to change these inequalities. Time in class decreased by more than the increase in non‐class learning time so that total learning time went down. However, these changes, especially those in class time, were similar for children from more and less well‐off families.

Where we do see a marginally significant increase in inequality is in learning time outside of class time. The estimate in column 6 suggests that a secondary school child from the 90^th^ family income percentile was engaging in about 30 minutes more learning (outside of online class time) than one from the 10^th^ percentile by family income.

In all, among secondary school students, there is no significant *worsening* in inequality in total learning time during lockdown, although children from better‐off households (as before lockdown) continued to spend significantly more time on learning activities than children from worse‐off households.

## V. Home‐learning resources and environment

The quality of home‐learning resources provided by schools and the quality of the home‐learning environment (study space, computer or tablet to access school resources) are likely to play an important role in determining how productive the time that children spend learning is for the accumulation of human capital. Moreover, better learning resources and environment may also make learning more interesting and enjoyable, possibly motivating children to do more of it. Complementarities between learning time and resources could create inequalities in human capital between those who do and those who do not have access to good learning support at home. Their role in determining inequalities may be especially important during the lockdown due to the rapid transition from learning mostly at school to learning exclusively at home; there is likely to be much larger heterogeneity in the degree of preparedness and availability of adequate support across families than across schools.

In this section, we describe inequalities in various key dimensions of home‐learning environments during lockdown. We consider the activities and resources that schools provided to replace school learning, access to digital technology that children can use to contact their teachers and complete their school work, and availability of a quiet dedicated space for learning at home. We then show suggestive evidence of complementarities between learning time and material investments, and we examine whether access to resources can, at least partly, explain the socio‐economic gradient of time spent learning during lockdown.

### 1. Variability in availability of home‐learning resources

**FIGURE 3:**
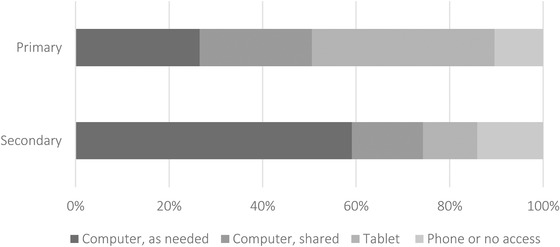
Distribution of access to technology *Note*: Calculated from responses to the survey question ‘What is the main device that [CHILD NAME] uses to access online resources provided by the school?’.

We start by examining the dispersion in access to technology across pupils in Figure 3.

Among primary school pupils, only around half had access to a computer for school (either their own or shared with someone else in the family). The most widely reported device was a tablet, used by 39 per cent of primary school students. One in ten students in primary school relied on a phone or had no device at all with which to access school work.

Secondary school students were more likely to have access to computers, especially their own computers. However, one in seven relied on a phone or had no device to access school work. As we show below, this may have been an especially binding constraint since online activities were more widespread in secondary schools (see Figure 5 later).

**FIGURE 4:**
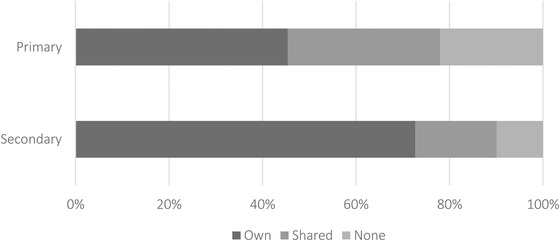
Distribution of access to a dedicated study space *Note*: Calculated from responses to the survey question ‘Does [CHILD NAME] have a desk or dedicated space for studying at home?’.

While access to technology and the internet has received a lot of media and policy attention as a potential barrier to productive home learning, much less has been said about availability of appropriate study space at home. Figure 4 shows that fewer than half of primary school students had their own dedicated space to study at home during lockdown and more than 20 per cent did not have access to any study space. At secondary school, this proportion is substantially smaller: 10 per cent of secondary school children did not have access to a dedicated study space during lockdown.

While home resources can help students make more effective use of their learning time, they are unlikely to substitute effectively for the professional teaching that children receive at school. One of the most striking features of school support for home learning during lockdown was how suddenly it was implemented, but the urgency to deliver led to fragmented and unequal provision. National guidance was thin on the ground, and largely left it to schools and even individual teachers to determine the aims of and resources for home learning among their students. Early studies from surveys of teachers such as TeacherTapp^26^ documented especially large differences between the resources provided by schools for older and younger children, and for those in the state and private sectors.^27^

**FIGURE 5:**
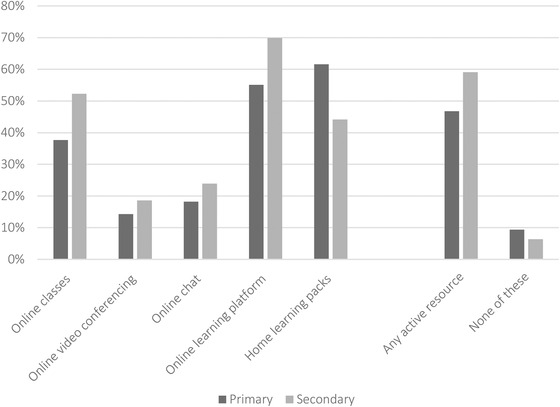
Home‐learning resources provided by schools *Note*: Calculated from responses to the survey question ‘Which of the following activities has [CHILD NAME]'s school provided while schools are closed? [Please tick all that apply]’.

To examine inequalities in school support, we asked parents about the resources that their child's school was providing, regardless of whether they were able to make use of them. Figure 5 shows the dispersion in school resource provision across students. Overall, 9 per cent and 6 per cent of primary and secondary school children, respectively, were not being offered any support through online classes, video or text chat, online learning platforms to set and collect work, or home‐learning packs at the time we administered the survey (i.e. 1–1.5 months after school closures). While this is a relatively small group of students, these children are likely to be significantly disadvantaged from their time in lockdown, without access to school resources to support their learning or maintain ties to their school.

On the other hand, just over half of students – 59 per cent of secondary and 47 per cent of primary students – were being offered some form of active learning (which includes online classes, video conferences or chats). Relatively few students were offered real‐time video conferencing or chatting with teachers. These resources can facilitate learning; they may also be helpful for students' social and emotional well‐being by helping to preserve connections with school, classmates and teachers.

### 2. Inequalities in home‐learning resources

A key question is whether the heterogeneity in school responses and home‐learning environments will widen inequalities between children from higher‐ and lower‐income families. To assess the likelihood of this, we first estimate the association between home‐learning resources and household income, regressing home‐learning resources on family income rank, controlling for the same set of covariates as in equation 1. For conciseness and to highlight the main margins of heterogeneity, we combine similar categories from Figures 3, 4 and 5 to construct the five outcome measures for this analysis presented in Table 3. For the resources provided by the school, we distinguish between ‘active resources’ (which include online classes, online video conferencing and online chat), ‘other resources’ (including online learning platforms, home‐learning packs and emails) and ‘none’ (when children received no support from school). The outcome variable in column 4 is an indicator for whether the child has access as needed to a computer or tablet for home learning, hence leaving out those who only occasionally have access to these. Finally, the outcome in column 5 is an indicator for whether the child has a dedicated study space, excluding those who share or have no space for learning activities.

**TABLE 3:** Socio‐economic gradient in school and home resources for learning

	*(1)*	*(2)*	*(3)*	*(4)*	*(5)*
	School resources				
	*Active resources*	*Other resources*	*None*	*Computer or tablet as needed*	*Own dedicated study space*
	*Panel A. Primary school students*				
Earnings rank	0.285***	−0.0917***	−0.0470***	0.0766**	0.186***
	(0.0505)	(0.0296)	(0.0176)	(0.0299)	(0.0521)
Observations	1,143	1,143	1,143	1,043	812
	*Panel B. Secondary school students*				
Earnings rank	0.126***	−0.0218	−0.0231	0.0656**	0.0907***
	(0.0417)	(0.0261)	(0.0153)	(0.0305)	(0.0302)
Observations	1,722	1,722	1,722	1,661	1,420

*Note*: ‘Active resources’ include online classes, video conferencing and online chat. ‘Other resources’ include online platforms, home‐learning packs, emails with information and other resources. ‘Computer or tablet as needed’ is an indicator that takes the value 1 if the child's main device to access school work is a computer or tablet that is always available or available most of the time, and the value 0 otherwise. ‘Own dedicated study space’ is an indicator that takes the value 1 if the child has a desk or dedicated space for studying for him/herself and 0 otherwise. All regressions also include a full set of age dummies, number of siblings, and indicators for whether the child lives in a lone‐parent household and whether he/she is the oldest. All regressions are weighted using the weights described in Section II. Standard errors are given in parentheses. ^***^ *p*<0.01, ^**^ *p*<0.05, ^*^ *p*<0.1.

Estimates in Table 3 show that income is strongly associated with the home‐learning resources that we measure in most cases. The income gradients are especially large for active resources provided by schools, which we would expect to be most productive for children's learning, and stronger for primary than for secondary school children. For example, a primary school child in the 10^th^ income percentile is 23 percentage points (or nearly 50 per cent relative to the overall mean) less likely to receive active school resources than a child in the 90^th^ income percentile; the equivalent parameter for secondary school children is 10 percentage points. In turn, better‐off children in primary school are less likely to receive other resources for learning from schools, or to attend schools that provide no resources at all, than their worse‐off peers. Moreover, better‐off families are significantly more likely to provide their children with the home resources needed for learning, including computer/tablet as needed and a desk of their own.

Our results in Section IV exposed significant levels of inequality in learning time by family income, which increased after lockdown for primary (but not for secondary) school children. The confinement of learning to the home and the inequalities in learning resources that we documented above may have compounded these inequalities. We now investigate this question further by asking whether, as we hypothesised above, there is a link between home‐learning resources and learning time and, if so, how much of the association between time spent learning and family income can be explained by the fact that children from better‐off families have better resources to support their learning.

Tables 4 and 5 show the results for primary and secondary school students respectively. For each of the three learning time categories studied before (total learning time, class time and non‐class time), we present estimates of their association with home‐learning resources provided by the school and available at home (first column), family income during lockdown (second column)^28^ and both sets of covariates (third column).

**TABLE 4:** Mediation analysis of learning time during the lockdown among primary school students

	*(1)*	*(2)*	*(3)*	*(4)*	*(5)*	*(6)*	*(7)*	*(8)*	*(9)*
	*Total learning time*			*Class time*			*Non‐class time*		
Earnings rank		2.063***	1.391***		1.610***	0.989***		0.749**	0.533*
		(0.290)	(0.285)		(0.191)	(0.178)		(0.291)	(0.296)
Active school resources	1.072***		0.964***	1.463***		1.383***	−0.00686		−0.0468
	(0.163)		(0.163)	(0.103)		(0.103)	(0.170)		(0.171)
Home study space (ref: no desk or dedicated study space)
Own desk/study space	1.447***		1.365***	0.412***		0.357***	1.314***		1.284***
	(0.220)		(0.218)	(0.139)		(0.138)	(0.229)		(0.229)
Shared desk/study space	0.569**		0.630***	−0.146		−0.106	0.810***		0.834***
	(0.238)		(0.236)	(0.151)		(0.149)	(0.247)		(0.247)
Computer or tablet available for studying (ref: no computer or tablet)
All or most of the time	0.895***		0.775***	0.709***		0.640***	0.214		0.167
	(0.299)		(0.297)	(0.188)		(0.186)	(0.311)		(0.312)
Rarely or some of the time	0.116		0.0687	0.338		0.325	−0.250		−0.270
	(0.387)		(0.383)	(0.243)		(0.240)	(0.403)		(0.403)
Constant	2.524***	3.733***	1.928***	0.439*	1.221***	−0.0125	2.106***	2.797***	1.883***
	(0.398)	(0.292)	(0.412)	(0.249)	(0.193)	(0.259)	(0.412)	(0.291)	(0.429)
Observations	1,056	1,057	1,056	1,097	1,097	1,097	1,065	1,066	1,065
R^2^	0.128	0.054	0.147	0.224	0.070	0.245	0.041	0.012	0.044

*Note*: Standard errors are given in parentheses. ^***^ *p*<0.01, ^**^ *p*<0.05, ^*^ *p*<0.1.

**TABLE 5:** Mediation analysis of learning time during the lockdown among secondary school students

	*(1)*	*(2)*	*(3)*	*(4)*	*(5)*	*(6)*	*(7)*	*(8)*	*(9)*
	*Total learning time*			*Class time*			*Non‐class time*		
Earnings rank		1.778***	1.627***		0.940***	0.702***		1.466***	1.465***
		(0.259)	(0.252)		(0.189)	(0.174)		(0.275)	(0.273)
Active school resources	1.359***		1.307***	1.718***		1.696***	0.0338		−0.00900
	(0.146)		(0.144)	(0.0997)		(0.0994)	(0.159)		(0.158)
Home study space (ref: no desk or dedicated study space)
Own desk/study space	0.667***		0.596**	0.220		0.186	0.674**		0.610**
	(0.253)		(0.250)	(0.170)		(0.170)	(0.276)		(0.274)
Shared desk/study space	0.566**		0.432	0.255		0.195	0.681**		0.560*
	(0.288)		(0.285)	(0.194)		(0.194)	(0.315)		(0.313)
Computer or tablet available for studying (ref: no computer or tablet)
All or most of the time	−0.0202		−0.0787	0.396***		0.371***	−0.235		−0.288
	(0.212)		(0.210)	(0.144)		(0.143)	(0.233)		(0.231)
Rarely or some of the time	0.614		0.711	0.0853		0.127	0.425		0.510
	(0.490)		(0.484)	(0.339)		(0.337)	(0.534)		(0.530)
Constant	2.709***	3.018***	1.959***	0.937***	1.934***	0.612**	1.608***	1.132***	0.944**
	(0.349)	(0.284)	(0.364)	(0.237)	(0.207)	(0.249)	(0.382)	(0.303)	(0.398)
Observations	1,562	1,562	1,562	1,631	1,631	1,631	1,594	1,595	1,594
R^2^	0.072	0.039	0.096	0.176	0.025	0.184	0.010	0.025	0.028

*Note*: Standard errors are given in parentheses. ^***^ *p*<0.01, ^**^ *p*<0.05, ^*^ *p*<0.1.

Columns 1, 4 and 7 in both tables show the relationship between learning resources and each category of learning time. Most of the home‐learning resources discussed above are positively associated with our measures of learning time. The provision of online classes or other active learning resources by the school is strongly positively correlated with class learning time for students in primary and secondary schools, but does not explain time spent on other learning activities. Having one's own desk is strongly positively correlated with both types of learning time for primary school students and with non‐class time for secondary school students. Finally, for both primary and secondary school students, having access to a computer/tablet all or most of the time is important for class time but not for non‐class time. Combined with evidence of an income gradient in time spent learning presented in Table 2 and in availability of home‐learning resources presented in Table 3, this evidence suggests that indeed the association between home‐learning time and income inequality is partly explained by the availability of home‐learning resources.

Results in the second and third columns for each outcome allow us to quantify the magnitude of the mediating power of the different home‐learning resources. At the primary school level, we see that adding controls for home‐learning resources reduces the size of the earnings rank coefficient by nearly a third for total learning time (compare estimates of the income gradient in columns 2 and 3 of Table 4), with similar reductions in specifications with class and non‐class time as outcomes (columns 5 and 6 and columns 8 and 9). At the secondary school level, home‐learning resources appear to be a less powerful mediator of the relationship between family income and learning time, reducing the association between total learning time and family income by about one‐tenth. It is possible that learning attitudes are more crystallised among older children, making their efforts less reliant on the resources they have available during this exceptional period. It is certainly also the case that we are not controlling for all types of resources that are important to support home learning, and this implies that we are likely underestimating the overall role of these resources in mediating the relationship between learning time and family income. It is possible that the omitted resources play a more important role, and hence that the bias is larger, at the secondary school level. Nevertheless, our results in columns 5 and 6 of Table 5 suggest that a quarter of the association between family income and *class* time is mediated by differences in learning resources, but almost none is mediated for the association between family income and *non‐class* time (columns 8 and 9).

To gauge the importance of the home‐learning resources in mediating the relationship between family income and learning time during lockdown, we implement the decomposition proposed by Gelbach (2016). This decomposition allows us to quantify the portions of the gap in learning time between poorer and better‐off children during lockdown that can be explained by availability of different home‐learning resources.^29^ In interpreting the results of the decomposition, it is worth highlighting that we are not controlling for some potentially important home‐learning resources (such as children's books and playing materials available at home) and this can bias estimates of our decomposition. Specifically, the omitted variables are likely to be positively related to the resources we are considering and result in upward‐biased estimates of the mediating effect of each home‐learning resource included in our model. However, and as mentioned before, our assessment of the *overall* mediating effect of home‐learning resources is likely to be downward biased since we are not considering all relevant dimensions of these resources.

For reference, the first row in Panel A of Table 6 reproduces the coefficients on earnings rank presented in columns 2, 5 and 8 of Table 4. Its exact interpretation is the change in hours spent on home learning associated with movement from the 1^st^ to 100^th^ family income percentile, or the learning time gap between the poorest and richest primary school children. The coefficients in lower rows show how much of that gap is explained by active support with home learning from the school, availability of home study space and availability of a computer or tablet. These three coefficients add up to the difference in the earnings rank coefficients in the second and third columns under each outcome in Table 4.

**TABLE 6:** Gelbach decomposition of learning time gaps between children from lower‐ and higher‐income families

	*(1)*	*(2)*	*(3)*
	*Total learning time*	*Class time*	*Non‐class time*
	*Panel A. Primary school students*		
Earnings rank	2.063***	1.610***	0.749**
	(0.290)	(0.191)	(0.291)
Active school resources	0.294***	0.431***	−0.0135
	(0.0716)	(0.0789)	(0.0496)
Home study space	0.272***	0.124***	0.191***
	(0.0762)	(0.0373)	(0.0717)
Computer or tablet availability	0.107**	0.0663**	0.0380
	(0.0433)	(0.0272)	(0.0350)
	*Panel B. Secondary school students*		
Earnings rank	1.778***	0.940***	1.466***
	(0.259)	(0.189)	(0.275)
Active school resources	0.144**	0.189**	−0.000898
	(0.0603)	(0.0747)	(0.0157)
Home study space	0.0331	0.0159	0.0407
	(0.0267)	(0.0159)	(0.0278)
Computer or tablet availability	−0.0257	0.0326*	−0.0389
	(0.0222)	(0.0178)	(0.0245)

*Note*: The coefficients for ‘Active school resources’, ‘Home study space’ and ‘Computer or tablet availability’ add up to the difference between the coefficients for ‘Earnings rank’ in the corresponding second and third columns of each panel in Tables 4 and 5 up to rounding error. Standard errors are given in parentheses. ^***^ *p*<0.01, ^**^ *p*<0.05, ^*^ *p*<0.1.

The coefficient of 0.294 for active school resources shows that the availability of active school resources explains about 14 per cent (0.294/2.063) of the lockdown learning time gap between the poorest and richest primary school students. We see that home study space explains a similar proportion, while availability of computers only about half of that. Combined variation in home resources explains only marginally (4 percentage points) more of the gap in total learning time between poorer and richer children than variation in support provided by schools. This suggests that decisions made by schools may have had an important role to play in determining home‐learning activities and inequalities therein during lockdown, of a similar order of magnitude to that of physical resources available at home. This evidence is reinforced by results for class time in column 2. There, we see that variation in the provision of active school resources explains over a quarter of the gap, more than twice as much as home study space and computer/tablet availability combined. But although its effect is smaller, having access to digital technology is also a significant driver of time spent learning in online classes for primary school children.

This is an important finding in light of anecdotal evidence that schools were hesitant to provide online support during lockdown in order to not disadvantage poorer children who do not have the home resources needed to access such support; for this reason, home‐learning packs were seen as potentially a more equitable home‐learning support tool. Our results provide some justification for this concern, but, since most children have access at least to a shared computer or tablet, they also show that choices made by the schools presented a more significant barrier to access to online learning (class time) for children from poorer backgrounds than lack of home resources.

As noted above, observed home‐learning resources are less good at explaining learning time gaps at secondary school level. Moreover, they only explain some of the family income gap in *class* (but not in non‐class) learning time among these children. Panel B of Table 6 shows that variation in provision of active school resources explains about a fifth of the gap in class learning time between the poorest and richest pupils, while the availability of a computer or tablet to support learning can only explain a modest 3 per cent of that gap. While still important, these figures suggest that learning attitudes and how they vary with the income of the family may already be crystallised among older children in ways that they are not among younger children, so that externally provided resources are less capable of influencing the learning behaviour of secondary school children.

## VI. Conclusions

The closure of schools in Spring 2020 in response to the COVID‐19 pandemic disrupted the daily lives and learning experiences of children. The transition period might have been particularly unsettling as families and schools had little time to prepare for new ways of delivering childcare and education activities to homebound children. The different choices made by families about home schooling and childcare provision and by schools about support for home learning during this time may have long‐lasting consequences for children's development and inequalities therein.

In this paper, we have examined children's time use during lockdown, focusing especially on learning time, with the aim of characterising children's learning experiences during lockdown and inferring, to the extent possible at this early stage, whether educational inequalities are likely to worsen in the longer run as the result of lockdown.

We have used a combination of existing data and novel data that we collected between 29 April and 20 June 2020, on 5,582 parents living in England with at least one child aged 4–15 and in year group Reception, 1, 4, 5, 8, 9 or 10. A key feature of our data is that they contain information on time use in one‐hour intervals over the course of the 24‐hour period preceding the survey. Although not collected using the same methodology as in specialised time use surveys due to logistical constraints, we show that our data compare well with such surveys, alleviating to a considerable degree concerns about excessive measurement error.

Our results offer compelling evidence to suggest that indeed inequalities may have worsened over the course of lockdown, especially for primary school students. We see that a considerable gap in learning time emerges between primary school children from poorer and better‐off families, which is not there prior to lockdown. In contrast, for secondary school pupils, inequalities in learning time persist over the course of lockdown but do not worsen relative to the pre‐lockdown period. Unsurprisingly, we find that poorer children live in homes where they are significantly less likely to have access to resources that are positively associated with learning time, including computers and/or tablets and dedicated study space. Perhaps less predictably, we also find that they had less access to active school support with home learning because their schools were less likely to provide them with support such as online classes, online video conferencing and online chat and more likely to support home learning through more passive means, such as assignment of learning packs.

Anecdotally, at least in part, the justification for this was a concern about inequity if poorer students would be less able to access ‘active’ learning support than richer students due to constraints in resources available at home, such as computers and internet access. However, decompositions of the learning time gap between poorer and richer pupils at primary school level show that variation in provision of active support by schools explains as much of the gap in home‐learning time between poorer and richer students as variation in availability of the home resources for learning that we measure, suggesting that school choices and/or constraints may have constituted an important driver of inequalities in learning during lockdown.

For primary school students, we are able to explain more of the gap between poorer and richer students with differences in physical resources available for home learning (both home and school provided) than for secondary school students (at 33 per cent and 8 per cent respectively). Combined with evidence of little overall change in the income gradient of learning time for secondary school students, this suggests that circumstances during lockdown may have played a more important role in the home‐learning experience of primary than secondary school students. We cannot rule out the possibility that, in fact, this is because the forms of support that we capture in the survey are more relevant for younger than older children and that other features of the home environment matter more for the latter group, such as parents' ability to help them with their work. However, it could also be that learning attitudes are more crystallised among older children, making their efforts less reliant on the resources available to them during this exceptional period.

At the time of writing, our findings suggest that there is a real risk that time spent learning at home since schools closed in March 2020 has widened educational inequalities between poorer and richer students, especially among primary school students. The ongoing risk of local or national spikes in COVID‐19 cases means that home learning may return during the academic year of 2020–21. If the pandemic forces schools to close again, it will continue particularly to deprive poorer students of the protective and (at least partly) equalising role that time in school can play for their learning and development.

The types of home‐learning support that the policy debate has focused on so far are important, but our findings strongly suggest that more coordination is required to prevent poorer students from falling behind. In the absence of coordination at the local and national levels, schools have responded to the crisis by offering markedly different packages of home‐learning materials. This has led to substantial inequalities along socio‐economic divides. There are potentially substantial benefits to developing and sharing resources across schools (as the Oak National Academy is doing). These will both improve equity in access to home learning and free up teachers' time to provide more individualised support to students. More broadly, there is a need for greater coordination between schools, local administrations and central government. Different schools serve different communities, so a coordinated response does not necessarily mean the same response everywhere. But there is a role for national policymakers in setting out a common set of guiding principles and aims, such as whether children should be expected to cover new material or to only consolidate knowledge while doing distance learning.

Our research further highlights, however, that ramping up efforts to equalise home‐learning experiences through the levers available to schools and policymakers is unlikely to improve the situation significantly as the dimensions of home learning that we measure explain only a relatively small proportion of the gap in learning between poorer and better‐off children. On the one hand, therefore, our results suggest that getting children back into school seems fairly imperative to tackle these growing inequalities; and on the other, there is a need for a much better understanding of the wide‐ranging effects of this shock on families and the complex ways in which these interact with pre‐existing inequalities in family circumstances.

We end on a note of caution. While the analysis presented is suggestive of increasing educational inequalities, more needs to be done before firmer conclusions can be made. Future work will link the survey data used in the analysis here to administrative data on children's school attainment before and after lockdown. This will allow us to study directly how lockdown will affect levels of attainment and inequalities therein, controlling for pre‐lockdown differences in school attainment and misbehaviour. The evidence presented so far, however, suggests that there is an urgent need for policies that not only support catch‐up at school among pupils who have fallen behind, but also streamline provision of school support over the course of what is likely to be a disrupted school year in 2020–21.

## Supporting information

• Appendix

## Footnotes

1 Furthermore, assuming 8–10 per cent returns to an additional year of education, Burgess and Sievertsen (2020) estimate a permanent impact on future earnings from missing one‐quarter of the school year to be about 2–2.5 per cent.

2 Hutchinson, 2016; Burgess and Sievertsen, 2020; Education Endowment Foundation, 2020.

3 Blundell et al., 2020.

4 Pischke, 2007.

5 See Steward, Watson and Campbell (2018) for an overview.

6 Marcotte and Hemelt, 2008; Marcotte and Hansen, 2010.

7 For example, Carneiro and Rodrigues (2009) and Attanasio et al. (2018 and 2020).

8 Hsin and Felfe, 2014; Fiorini and Keane, 2014; Del Bono et al., 2016.

9 Del Bono et al., 2016.

10 Rai, 2015.

11 For example, Aizer, Stroud and Buka (2016) and Persson and Rossin‐Slater (2018).

12 Crnic, Greenberg and Slough, 1986.

13 For example, Gutman, Vonnie and Teru (2005) and Oreopoulos, Page and Stevens (2008).

14 Banks and Xu, 2020; Henderson et al., 2020.

15 Gershuny and Sullivan, 2017.

16 In England, parents are statutorily obliged to send their child to school from the school term that begins after the child's fifth birthday. However, schools have discretion to admit children earlier than this, and almost all children in England are in Reception class from September of the year in which they turn 4.

17 Data for 90 per cent of the sample were collected before 15 May 2020.

18 More information on the UKTUS can be found in Gershuny and Sullivan (2017).

19 We use standard procedures to equivalise earnings and count the first adult as one member, subsequent adults and children aged 14 and over as half an equivalent member, and younger children as 0.3 of an equivalent member.

20 We treat outliers in the following way. We set to missing any observations reporting weekly hours above the 95^th^ percentile of the distribution of each of the four measures. We divide the weekly information in our survey by 5 to measure time spent on those activities per day so that the measure for our survey is comparable to the measure from the UKTUS. To construct total learning time, we add the time spent on each of the four activities daily. We set any observation reporting more than 12 hours of learning per day to 12.

21 There may be a concern that these data would be noisier than the time‐diary data we have presented so far as they are collected in a more aggregated way. However, Table A2 in the online appendix shows that the average amount of time spent on learning activities per day based on our time‐diary data is remarkably similar to that based on the weekly recall data.

22 Hsin and Felfe, 2014; Fiorini and Keane, 2014; Del Bono et al., 2016.

23 Attanasio, 2015.

24 We also ran the analysis not conditional on this vector of child and household characteristics. The results were very similar and are not included in the paper, but are available upon request.

25 Specifically, in UKTUS, we categorise activities referring to ‘classes and lectures’ (activity code 2110) and ‘unspecified study’, defined as ‘studies at primary, secondary and tertiary education institutions as part of the formal education system, including general and vocational training’ (activity code 2000).

26 For example, see https://teachertapp.co.uk/what-does-distance-learning-look-like-in-england-and-where-will-teachers-kids-be-today/.

27 In published analysis using these data, Andrew et al. (2020) find similar patterns, with private schools much more likely to offer online resources to their students. However, the authors show that substantial inequalities exist even within the state school sector.

28 The coefficient on earnings rank in this column could be slightly different from the sum of the coefficients in the second and third rows of Table 2. This is because, while the two regressions condition on the same covariates (child's age, dummy for lone‐parent household, dummy for the child being oldest, and the number of siblings in the household), we do not include interactions between these covariates and the ‘Post’ dummy in equation 1, whose estimates are reported in Table 2.

29 The Gelbach method decomposes the difference between estimators from an unconditional linear model of the effect of earnings on learning time and a conditional model that includes a set of mediators, using a simple omitted variable bias formula. It is implemented in the following way. (1) The unconditional estimator comes from the linear model $Y=\alpha +\beta_{1}^{U}Income+\varepsilon$. (2) The conditional estimator comes from a model that includes a set of k (in our case three) mediators, M: $Y=\alpha +\beta_{1}^{C}Income+M\beta_{2}+\varepsilon$. The Gelbach decomposition of the difference between $\beta_{1}^{U}$ and $\beta_{1}^{C}$ is as follows: $\delta =\beta_{1}^{U}-\beta_{1}^{C}=\vartheta^{1}\beta_{2}^{1}+\cdots +\vartheta^{k}\beta_{2}^{k}=\sum_{j=1}^{k}\vartheta^{j}\beta_{2}^{j}$, where $\vartheta^{j}$ is the coefficient for mediator *j*, obtained from regressing $M_{j}$ on Income, and $\delta_{j}=\vartheta^{j}\beta_{2}^{j}$ is the component of the difference *δ* due to mediator *j*.
