# Supplementary material for: Inequalities in Children's Experiences of Home Learning during the COVID‐19 Lockdown in England
Source: Fisc Stud. 2020 Nov 30;41(3):653–83. doi: 10.1111/1475-5890.12240 (PMC7753283; doi:10.1111/1475-5890.12240)
Supplement: Supplementary file 1 — • Appendix [file FISC-41-653-s001.pdf]

Appendix. Extra tables and figure

Table A1. Means for our survey sample (weighted and unweighted) compared with nationally representative LFS sample

	IFS–IoE survey, unweighted	IFS–IoE survey, reweighted	Comparable LFS sample
Family structure			
Single mother	0.184	0.244	0.222
Single father	0.079	0.022	0.017
Couple	0.737	0.734	0.761
Women's education			
GCSEs or less	0.265	0.339	0.367
A levels	0.310	0.262	0.249
University degree	0.425	0.398	0.384
Men's education			
GCSEs or less	0.306	0.376	0.416
A levels	0.259	0.230	0.229
University degree	0.435	0.393	0.354
Single mothers' education			
GCSEs or less	0.358	0.441	0.495
A levels	0.423	0.308	0.272
University degree	0.219	0.251	0.233
Pre-crisis employment			
Women	0.728	0.752	0.745
Men	0.877	0.919	0.935
Single mothers	0.732	0.700	0.678
Women's pre-crisis earnings			
£0–£9,999	0.306	0.455	0.476
£10,000–£24,999	0.427	0.290	0.285
£25,000–£39,999	0.128	0.153	0.151
£40,000+	0.139	0.102	0.089
Men's pre-crisis earnings			
£0–£9,999	0.095	0.135	0.131
£10,000–£24,999	0.338	0.211	0.206
£25,000–£39,999	0.251	0.305	0.301
£40,000–£59,999	0.163	0.187	0.188
£60,000+	0.153	0.162	0.174
Single mothers' pre-crisis earnings			
£0–£9,999	0.300	0.556	0.594
£10,000–£24,999	0.521	0.283	0.256
£25,000–£39,999	0.084	0.122	0.115
£40,000+	0.094	0.039	0.035
Proportion working pre-crisis in industry where 50%+ of jobs have been locked down			
Women	0.330	0.255	0.231
Men	0.331	0.287	0.264
Single mothers	0.394	0.323	0.282
Proportion working pre-crisis in occupation where home working is possible in 0–15% of jobs			
Women	0.314	0.327	0.327
Men	0.347	0.351	0.362
Single mothers	0.351	0.379	0.392

	IFS–IoE survey, unweighted	IFS–IoE survey, reweighted	Comparable LFS sample
Proportion working pre-crisis in occupation where home working is possible in 15.1–75% of jobs			
Women	0.211	0.217	0.237
Men	0.271	0.212	0.192
Single mothers	0.228	0.272	0.300
Proportion working pre-crisis in occupation where home working is possible in 75.1–100% of jobs			
Women	0.474	0.456	0.436
Men	0.382	0.437	0.445
Single mothers	0.421	0.349	0.309
Region			
East Midlands	0.079	0.087	0.092
East of England	0.071	0.050	0.041
Greater London	0.184	0.125	0.118
North West	0.145	0.143	0.136
North East	0.072	0.065	0.061
South East	0.148	0.211	0.235
South West	0.102	0.104	0.097
West Midlands	0.109	0.111	0.107
Yorkshire and the Humber	0.091	0.105	0.113

Note: In order to make our analysis representative of the situation in England as a whole, we reweight our sample by key characteristics to ensure that it better matches the distribution of characteristics observed in the LFS. In particular, we reweight on: family structure, women's education, men's education, prior (pre-pandemic) employment, women's 2019 pre-tax earnings, men's 2019 pre-tax earnings and geographic region. To do this, we pool our data with the LFS sample and use regression analysis to calculate appropriate weights. We truncate our weights at the 10th and 90th percentiles to prevent our analysis being overly sensitive to a few observations. This table reports the means of the variables in the LFS comparable sample and in our survey sample, before and after being weighted.

Source: Labour Force Survey 2019 and COVID-19 online survey of families with children.

Figure A1. **Children's and adolescents' daily activities, 2014–15: one-hour compared with 10-minute intervals**

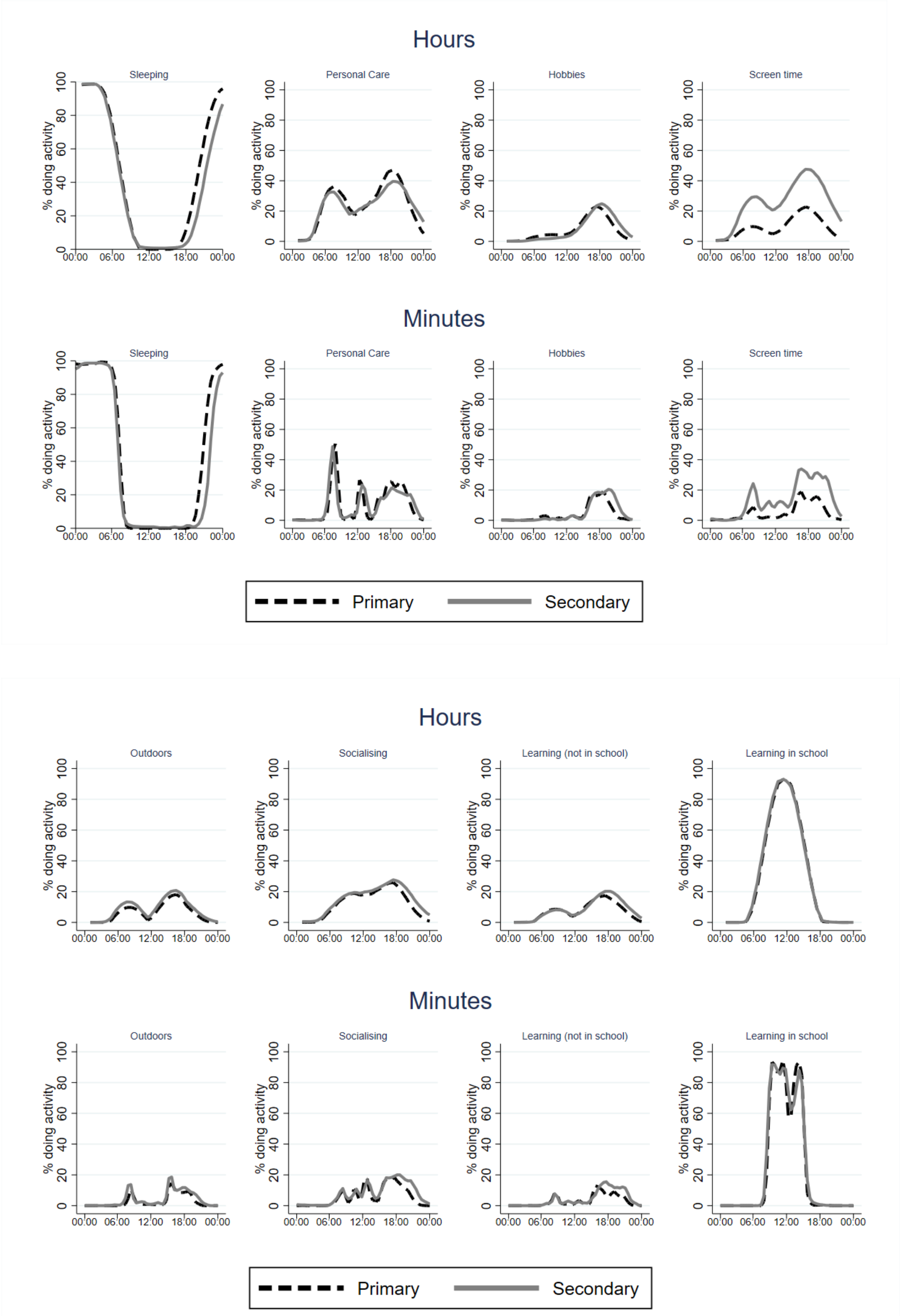

Note: Lines show the percentage of individuals reporting doing each activity in each hour and each 10-minute slot of the day for the UKTUS (2014–15). All lines are smoothed using Epanechnikov kernels. For the hourly plots, each activity is coded as having taken place if it is recorded to have happened in any 10-minute slot in that hour.

Table A2. Different ways of measuring time spent on learning activities on a ‘school’ day during lockdown

	Primary	Secondary	Total
One-hour slots (time-diary data)	4.91	4.58	4.8
Parental recall of learning time	4.06	4.15	4.1
